# A Novel Illumination Compensation Technique for Multi-Spectral Imaging in NDVI Detection

**DOI:** 10.3390/s19081859

**Published:** 2019-04-18

**Authors:** Rui Jiang, Pei Wang, Yan Xu, Zhiyan Zhou, Xiwen Luo, Yubin Lan

**Affiliations:** 1 College of Engineering, South China Agricultural University/Guangdong Engineering Research Center for Agricultural Aviation Application (ERCAAA), Guangzhou 510642, China; jayzeve@126.com (R.J.); wangpei@scau.edu.cn (P.W.); autoxuyan@stu.scau.edu.cn (Y.X.); xwluo@scau.edu.cn (X.L.); ylan@scau.edu.cn (Y.L.); 2 National Center for International Collaboration Research on Precision Agricultural Aviation Pesticides Spraying Technology (NPAAC), Guangzhou 510642, China; 3 Key Laboratory of Key Technology on Agricultural Machine and Equipment, South China Agricultural University, Ministry of Education, Guangzhou 510642, China; 4 School of Electrical and Information Engineering, Jiangsu University, Zhenjiang 212013, China

**Keywords:** active illumination compensation, remote sensing, vegetation index, multi-spectral imaging, NDVI, light attenuation model

## Abstract

To overcome the dependence on sunlight of multi-spectral cameras, an active light source multi-spectral imaging system was designed and a preliminary experimental study was conducted at night without solar interference. The system includes an active light source and a multi-spectral camera. The active light source consists of four integrated LED (Light Emitting Diode) arrays and adjustable constant current power supplies. The red LED arrays and the near-infrared LED arrays are each driven by an independently adjustable constant current power supply. The center wavelengths of the light source are 668 nm and 840 nm, which are consistent with that of filter lens of the Rededge-M multi-spectral camera. This paper shows that the radiation intensity measured is proportional to the drive current and is inversely proportional to the radiation distance, which is in accordance with the inverse square law of light. Taking the inverse square law of light into account, a radiation attenuation model was established based on the principle of image system and spatial geometry theory. After a verification test of the radiation attenuation model, it can be concluded that the average error between the radiation intensity obtained using this model and the actual measured value using a spectrometer is less than 0.0003 w/m^2^. In addition, the fitting curve of the multi-spectral image grayscale digital number (DN) and reflected radiation intensity at the 668 nm (Red light) is *y* = −3484230*x*^2^ + 721083*x* + 5558, with a determination coefficient of R^2^ = 0.998. The fitting curve with the 840 nm (near-infrared light) is *y* = 491469.88*x* + 3204, with a determination coefficient of R^2^ = 0.995, so the reflected radiation intensity on the plant canopy can be calculated according to the grayscale DN. Finally, the reflectance of red light and near-infrared light can be calculated, as well as the Normalized Difference Vegetation Index (NDVI) index. Based on the above model, four plants were placed at 2.85 m away from the active light source multi-spectral imaging system for testing. Meanwhile, NDVI index of each plant was measured by a Greenseeker hand-held crop sensor. The results show that the data from the two systems were linearly related and correlated with a coefficient of 0.995, indicating that the system in this article can effectively detect the vegetation NDVI index. If we want to use this technology for remote sensing in UAV, the radiation intensity attenuation and working distance of the light source are issues that need to be considered carefully.

## 1. Introduction

The Normalized Difference Vegetation Index (NDVI) crop growth parameter is an important indicator of crop growth status [1]. Rapid, non-destructive and accurate monitoring of the crop nitrogen status is important for diagnosing crop growth characteristics, improving the nitrogen management level and utilization efficiency, and reducing farmland environmental pollution brought by excessive nitrogen application [2,3,4]. It is of great significance to further promote precision agriculture and digital agriculture [5,6,7]. Canopy spectral sensing technology based on remote sensing sensors [8] is an important means to obtain crop growth information indicators [9,10]. Remote sensing sensors are divided into active light source sensors and passive light source sensors according to their working methods, and they are called active sensors and passive sensors respectively [11]. Passive sensors receive solar radiation reflected by the vegetation or their own thermal radiation energy [12]. However, since the passive light source measuring instrument mainly regards sunlight as a radiation source, its greatest limitation is that it is easily affected by insufficient sunlight intensity and the solar zenith angle. Therefore, it generally needs to be used when the weather is clear and using a small solar zenith angle [13,14,15].

Researchers have obviously begun to think about solving the problems and disadvantages of passive sensors [16]. To overcome the limitation of passive sensors being susceptible to light conditions, several sensors based on spectral principles and active light sources have appeared in many countries [17]. These devices use a software difference algorithm to remove the influence of sunlight. Ding et al. [18,19] from the National Agricultural Information Engineering Technology Center at Nanjing Agricultural University designed an active light source canopy reflectance spectrometer, and its stability was controlled well. A narrow-band LED was used as the active light source to emit a high-frequency pulsed light. A photoelectric induction circuit receives crop canopy reflectance spectrum to calculate vegetation index. However, existing active sensors have a small detection area and require close-range detection, it is difficult to obtain large-scale macro-vegetation index information [20,21].

Currently, some active light source canopy-scale nitrogen measurement sensors are available for commercial use, such as GreenSeeker handheld crop sensor (Trimble Inc., Sunnyvale, CA, USA), CropCircle™ sensor (Holland Scientific Inc., Lincoln, NC, USA), N-Sensor ALS (Yara Inc., Oslo, Norway) and Crop Spec (Topcon Inc., Tokyo, Japan) [22,23]. These products equipped with active light sources that are not subject to ambient light conditions and can work on cloudy days or at night. In addition, these instruments have the advantages of high measurement accuracy and good data reliability, but they still have issues, such as complicated operation and small coverage area [24]. Since these devices need to sweep point by point on the crop canopy, so the obtained vegetation index makes it difficult to characterize the overall growth of the crop in a large area [4]. Usually, a large number of collection points needs to be distributed in the field for data collection, and the efficiency of these kinds of active sensors is relatively low. Multi-spectral camera technology has developed rapidly in recent years with better controllability and lower costs [25,26,27]. However, at present, the small-sized, lightweight multi-spectral camera still uses sunlight as its light source, and it is still unable to operate normally when the sunlight intensity is insufficient and the solar zenith angle is big. Therefore, it’s necessary to expand the operating time window of multi-spectral camera. Related researches about combination of multi-spectral camera and active light source have not been reported. Considering the unique advantages of active light source remote sensing and of multi-spectral camera, this paper intends to combine the multi-spectral camera with active light source technology to design an active remote sensing system for increasing the working time window of Remote Sensing.

## 2. Materials

### 2.1. GreenSeeker Handheld Crop Sensor

The Greenseeker hand-held crop sensor manufactured by Trimble Navigation Company in the United States has two independent light-emitting diodes, which emit near infrared light at 774 nm and red light at 656 nm, respectively [28]. The light emitting diode emits 40 kHz pulsed light, and then captures two wavelengths of reflected light on the surface of the object through a silicon photodiode detector. The instrument is often used to calculate the normalized vegetation index NDVI _(656,774)_:

(1) $$\mathrm{NDVI}{\;}_{\left(656,774\right)}=\frac{R774-R656}{R774+R656}$$

where *R774* is the reflectance of the near infrared light at 774 nm and *R656* is the reflectance of the red light at 656 nm.

### 2.2. Multi-Spectral Camera

**Figure 1:**
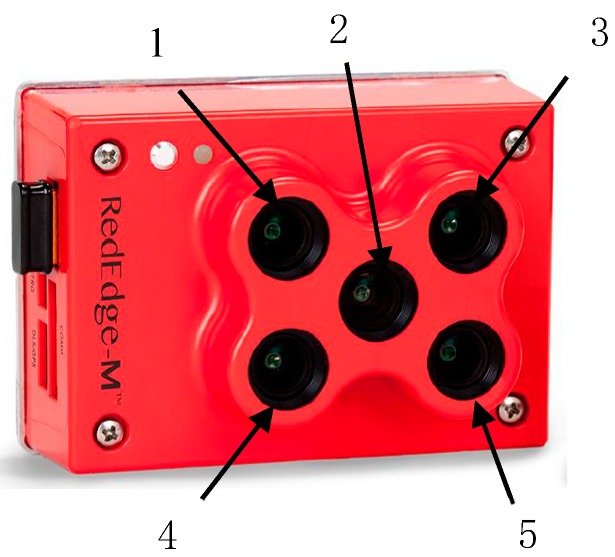
Rededge- M multi-spectral camera. Note: 1. Blue band 2. Green band 3. Red band 4. NIR (near infrared red) band 5. Red Edge band.

The RedEdge-M camera is a multi-spectral snapshot camera produced by the Micasense Company (Seattle, WA, USA). As shown in Figure 1, it is a multi-spectral imaging device that is advanced, light and compact and can be mounted on UAVs. It provides accurate multi-spectral data for agricultural remote sensing and has five independent imagers. Custom narrowband filters allow each imager to receive spectra in an accurate wavelength range. Table 1 lists the main specifications of the camera.

The Rededge-M multi-spectral camera has five independent CMOS sensors, which can acquire information of five spectral passbands at the same time. Table 2 shows the spectral passband parameters of the multi-spectral camera.

In this paper, only red (668 nm) with 10 nm of full width at half maximum (FWHM) and NIR (840 nm) with 40 nm (FWHM) were used.

### 2.3. Active Light Source

#### 2.3.1. Integrated LED Array

The normalized difference vegetation index (NDVI) is one of the most important parameters reflecting the crop growth and nutrition information [29]. According to this parameter, crop growth can be judged effectively and fertilization can be guided. The equation is:

(2) $$\mathrm{NDVI}{\;}_{\left(668,840\right)}=\frac{\frac{NIR}{NIR_{1}}-\frac{R}{R_{1}}}{\frac{NIR}{NIR_{1}}+\frac{R}{R_{1}}}$$

in which:

*NIR* is the reflection intensity of near-infrared light, w/m^2^.
*NIR_1_* is the incident intensity of near-infrared light, w/m^2^.
*R* is the reflection intensity of red light, w/m^2^.
*R*_1_ is the incident intensity of red light, w/m^2^.

**Figure 2:**
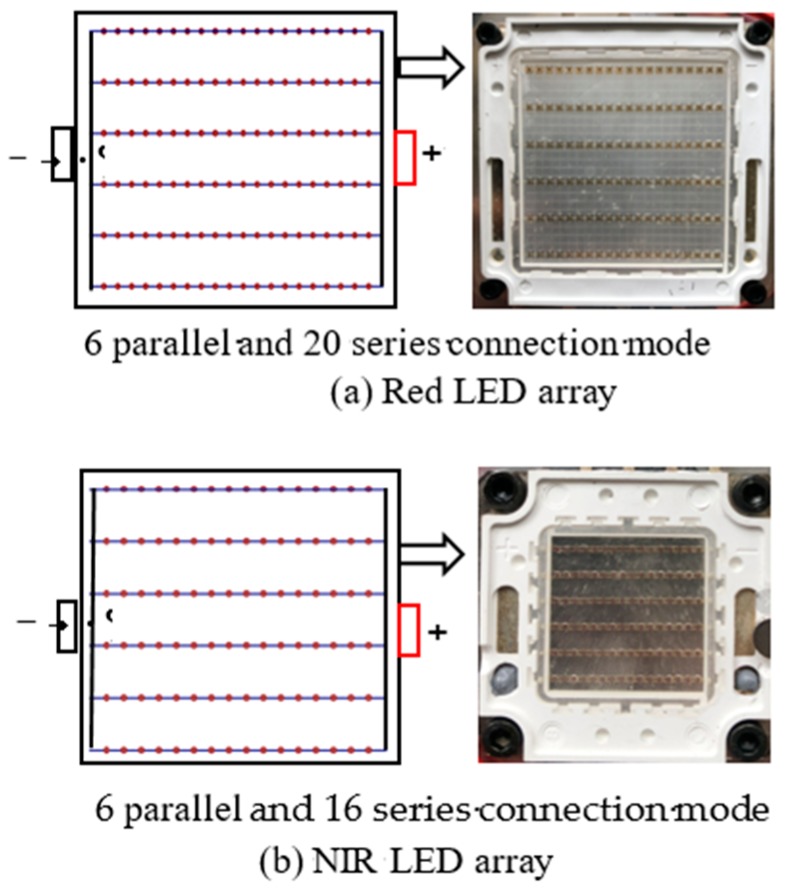
The active light source of the LED array.

Because the red central wavelength of Rededge-M multi-spectral camera is 668 nm and the center wavelength of the near-infrared light is 840 nm, the active light sources need to have the same wavelengths. In this study, an integrated LED arrays were used as shown in Figure 2. The two red LED arrays adopted 6 parallel and 20 series electrical connection mode of which the maximum drive current and voltage are 1800 mA and 32 V, respectively. Two NIR LED arrays adopted 6 parallel and 16 series electrical connection modes that have a maximum drive current and voltage of 1000 mA and 32 V, respectively.

**Figure 3:**
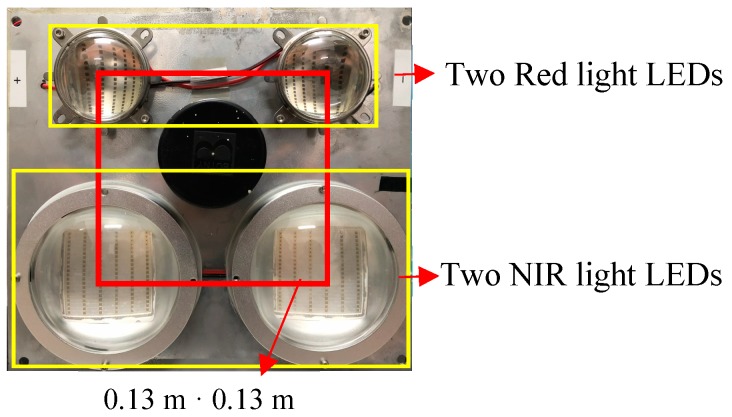
Active light source.

Considering that the radiation intensity of light decreases with the increase of distance, it is necessary to form a relatively uniform radiation intensity spot in the circle coinciding with the center of the camera lens in order to calibrate the radiation of the photographs taken by the multi-spectral camera. Therefore, the two groups of red light sources and near infrared light sources need to be symmetrical, as shown in Figure 3. Because there is no lens in front of the LED arrays, the light it emits is a uniform non-parallel light source, which is a kind of light similar to the sphere.

#### 2.3.2. Constant Current Power Supply

**Figure 4:**
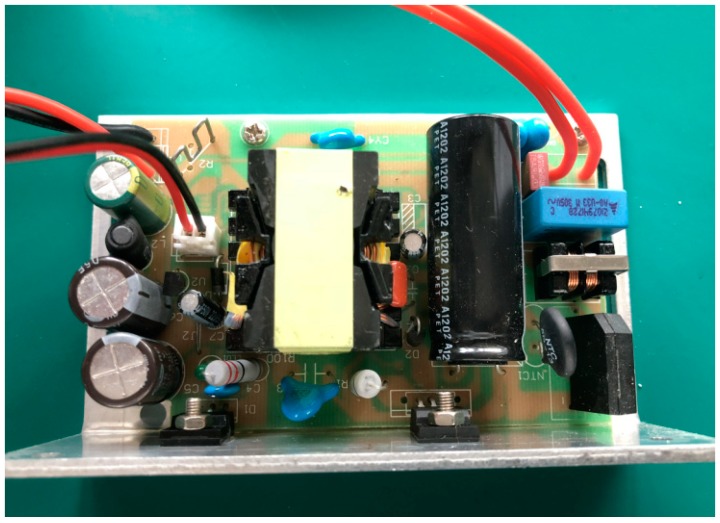
Adjustable constant current power supply.

The driving current of integrated LED array is more than 1000 mA and the voltage is as high as 32 V. The driving current of constant current power supply is only about 300 mA, so this active light source cannot drive normally. OB2269 is a low power consumption, noise-free, high conversion efficiency constant current driver chip, produced by On-Bright Electronics, Shanghai, China. A constant current drive power supply based on the chip is applied, as shown in Figure 4. The main parameters of constant current power supply are shown in Table 3, and all parameters can be controlled.

## 3. Methods and Results

### 3.1. Radiation Attenuation Test and Analysis

To accurately measure the spectral characteristics of the active light source, the EU2000+ spectrometer manufactured by Hangzhou Tongshang Optoelectronics Co., Ltd. (Hangzhou, China) was used to test the active light source. The EU2000 + spectrometer uses grating to separate light source, and then uses high sensitivity CCD arrays with 2048 units to measure. Its measurement range and wavelength resolution fully meet the accuracy requirements of this study. Table 4 shows the main specifications of the EU2000+ spectrometer.

**Figure 5:**
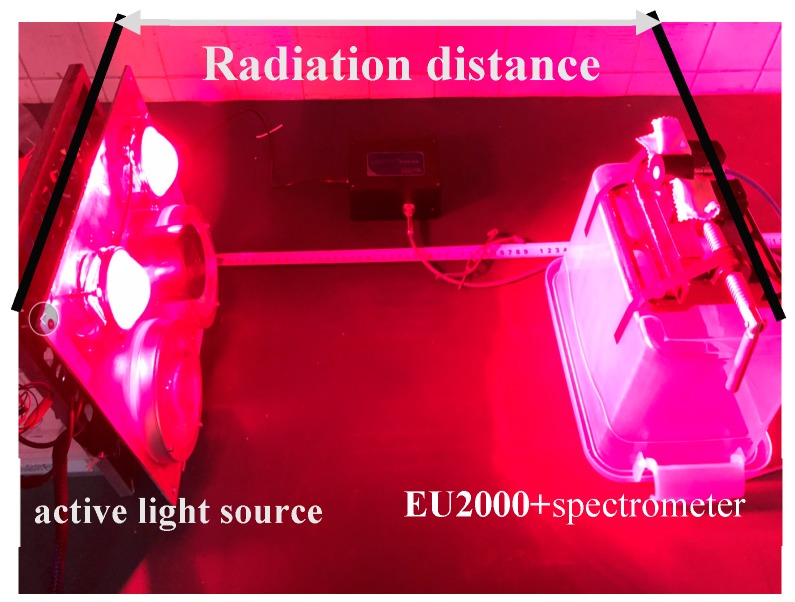
Spectral attenuation test site.

**Figure 6:**
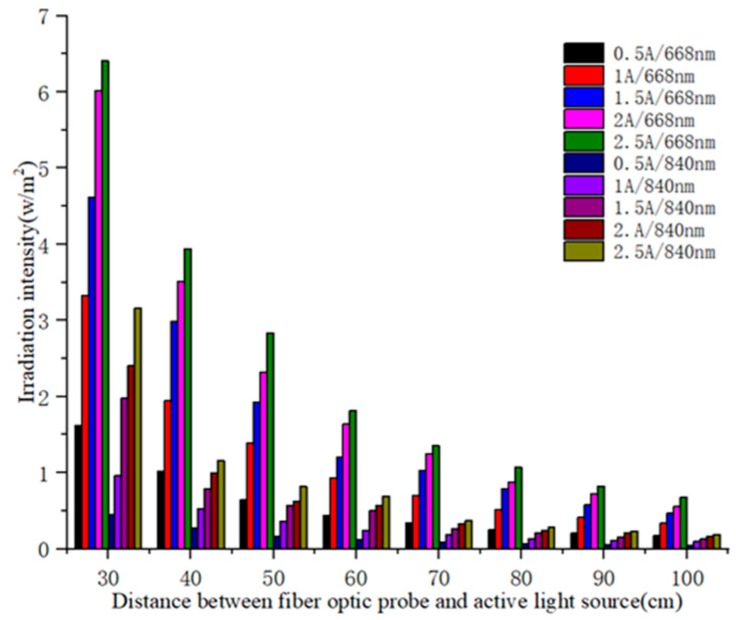
Relationship between the irradiation intensity and driving current and projection distance.

In this section, the EU2000 + spectrometer is used to measure the radiation intensity of the active light source. Two red LED arrays of active light source and two near infrared LED arrays are in parallel mode. Two bands of LED arrays are driven by two constant current power sources. The test location is shown in Figure 5. The driving current is set to 0.5, 1, 1.5, 2 and 2.5A in turn. When each current drives the light source, the distance between the probe and the light source of the spectrometer is changed from 0.3, 0.4, 0.5, 0.9 and 1.0 m, respectively. The results are shown in Figure 6.

From Figure 6, we can see that the radiation intensity of the active light source is proportional to the driving current and inversely proportional to the distance between the optical fiber probe and the active light source. As shown in Table 5, the radiation intensity ratios of 668 nm red light and 840 nm near red light are close to 4 under different driving currents of 30 cm and 60 cm, 40 cm and 80 cm, 50 cm and 100 cm radiation distances, which conform to the square inverse ratio law of light [30]. That is, when the measuring distance is doubled, the light intensity is reduced by a factor of four. Therefore, the radiation intensity on the surface of an object is negatively exponentially with increasing distance. The result is calculated as follows:

(3) $$L_{ec}=\frac{L_{e}}{D^{2}}$$

in which: *L_ec_* is the incident radiation intensity reaching the surface of the object, w/m^2^; *Le* is the emitted light radiation intensity for one LED array source, w/m^2^ and *D* indicates the ratio of the distance between the back and the distance measured for the first time.

### 3.2. The Establishment of Radiation Attenuation Correction Model

**Figure 7:**
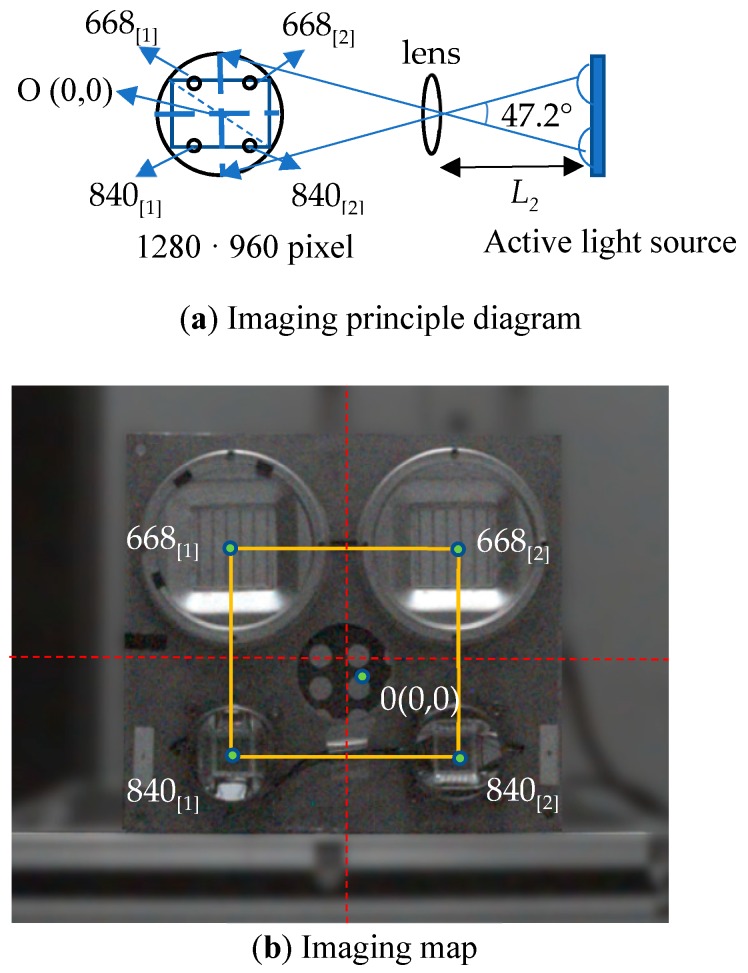
Imaging principle diagram.

As we have seen before, the intensity of radiation varies with the distance to the radiation source. It is necessary to establish a radiation attenuation model for calculating the radiation intensity on the surface of an object in real time according to the radiation distance. There are three steps to implement it. In the first step, the center axis of the multi-spectral camera is the same as that of the active light source, and the distance between the active light source and the multi-spectral camera is set to *L*_2_ = 1 m. In the second step, multi-spectral camera is used to directly face the active light source and take pictures. Finally, the coordinates of the four central points of the LED array in the multi-spectral image are obtained from the image using the center point O (0,0) as the coordinate origin. The imaging principle is shown in Figure 7a and the spectral image is shown in Figure 7b.

When the distance *L*_2_ is 1 m, the pixel coordinates of the four light sources in the image are 668_[1]_(−91,91), 668_[2]_(91,91), 840_[1]_(−91,−91), and 840_[2]_(91,−91).

As *L*_2_ gradually becomes larger, the center position of the four light sources in the image will be changed and the coordinates of 668_[1]_, 668_[2]_, 840_[1]_, and 840_[2]_ will gradually approach the center point O (0,0). The mathematical relationships between radiation intensity and distance are shown in Table 6. According to the pixel coordinates, the radiation intensity can be expressed by the pixel coordinates of four light sources in the image.

**Figure 8:**
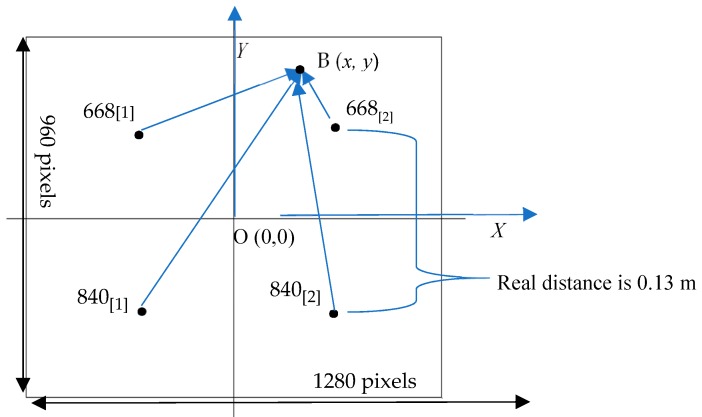
Distribution of the radiation intensity of incident light in multi-spectral images.

Different points in the new image represent different radiation intensities. In order to accurately obtain the radiation intensity of specific test points in the image, it is necessary to correct the radiation intensity at different locations. Among them, the radiation intensity of four light sources is only related to l2, but the radiation intensity of other points in the image needs geometric correction. It is certain that when the distance increases, the coordinates of the four light sources in the image and other coordinates (such as B (*x*,*y*)) in the image will change. Assume that B (*x*,*y*) is the test point in the first quadrant of the image, as shown in Figure 8.

Since the four light sources form a square of 0.13 m × 0.13 m, the actual distance represented by each pixel is:

(4) $$D_{X}=\frac{0.13}{91*2}\approx 0.00071\;m$$

In the first quadrant, point B receives radiation from four sources: 668_[1]_, 668_[2]_, 840_[1]_, and 840_[2]_. According to Equation (3) and space geometry, the radiation intensity of two 668 nm red LEDs at point B is as follows:

(5) $$L_{ec}668_{\left[1\right]}\;B=\frac{L_{e}668}{L_{2}{}^{2}+\left({\left[\left(\left|x\right|+91\right)*D_{X}\right]}^{2}+{\left[\left(\left|y\right|-91\right)*D_{X}\right]}^{2}\right)}$$

(6) $$L_{ec}668_{\left[2\right]}\;B=\frac{L_{e}668}{L_{2}{}^{2}+\left({\left[\left(91-\left|x\right|\right)*D_{X}\right]}^{2}+{\left[\left(\left|y\right|-91\right)*D_{X}\right]}^{2}\right)}$$

Similarly, the radiation intensity of two 840 nm NIR LEDs at point B is:

(7) $$L_{ec}840_{\left[1\right]}\;B=\frac{L_{e}840}{L_{2}{}^{2}+\left({\left[\left(\left|x\right|+91\right)*D_{X}\right]}^{2}+{\left[\left(\left|y\right|+91\right)*D_{X}\right]}^{2}\right)}$$

(8) $$L_{ec}840_{\left[2\right]}\;B=\frac{L_{e}840}{L_{2}{}^{2}+\left({\left[\left(91-\left|x\right|\right)*D_{X}\right]}^{2}+{\left[\left(\left|y\right|+91\right)*D_{X}\right]}^{2}\right)}$$

Therefore, the total radiation intensity of red and infrared light at point B is as follows:

(9) $$L_{ec}668\;B=L_{ec}668_{\left[1\right]}\;B+L_{ec}668_{\left[2\right]}\;B$$

(10) $$L_{ec}840\;B=L_{ec}840_{\left[1\right]}\;B+L_{ec}840_{\left[2\right]}\;B$$

Different quadrants have different calculation Equations, so it is necessary to calculate D_X_ in actual test.

### 3.3. Optical Radiation Attenuation Correction Model Verification

In order to further determine the correctness of the optical attenuation calibration model and ensure the successful application of the combination of active light source and multi-spectral camera, the validation experiments were carried out.

When the driving currents are all set to 1.5A, the actual radiation intensity of each LED array at a distance of 1 m from the active light source is measured by EU2000 + spectrometer and the measured radiation intensity are denoted as either *Le668* and *Le840*. After measuring, the radiation intensity of *Le*668 was 0.0469 w/m^2^ and *Le*840 was 0.0417 w/m^2^ when the distance is 1 m. When the distance is 2 m, the *D_x_* = 0.00035 m and coordinates of 668_[1]_, 668_[2]_, 840_[1]_, and 840_[2]_ in image were (45.5,45.5), (−45.5,45.5), (−45.5,−45.5) and (45.5,−45.5), respectively.

**Figure 9:**
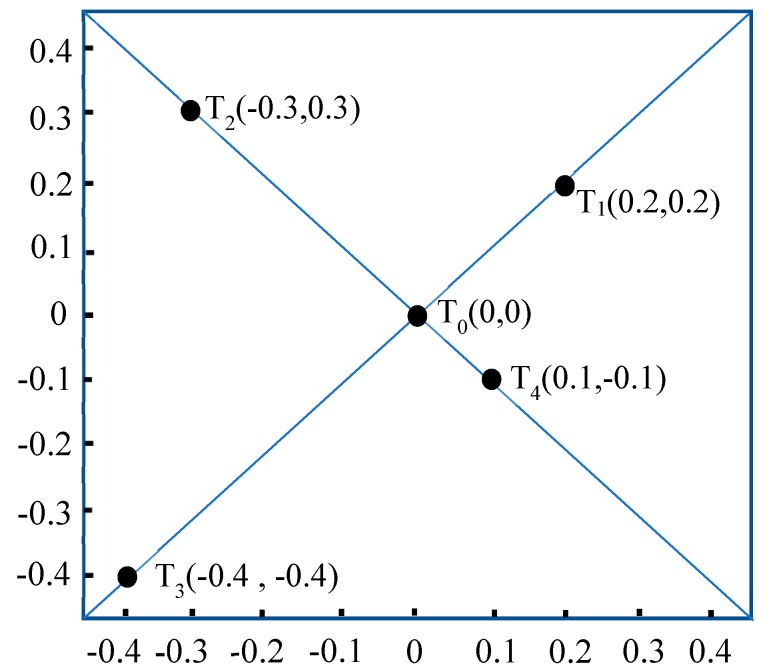
Optical attenuation correction model verification panel.

The Rededge-M camera is placed in the central circular hole of the active light source, and a square test board with a side length of 0.9 m is placed 2 m away from the active light source. The test board is marked with marked with T_0_ (0,0), T_1_ (0.2 m,0.2 m), T_2_ (−0.3 m,0.3 m), T_3_ (−0.4 m,−0.4 m), and T_4_ (0.1 m,−0.1 m) for a total of five test points as shown in Figure 9. Based on the previous measurements of *Le668* and *Le840* as well as Equations (9) and (10).

We can calculate the radiation intensity of five points in the image. In the meantime, actual test radiation intensity was measured at the above five points using an EU2000+ spectrometer. The results were listed in Table 7.

The results show that when the distance between the object and the camera is known, the error between the measured radiation intensity of the object and the calculated value based on the established radiation correction model is less than 0.0003W/m^2^, and the effect is acceptable.

### 3.4. Grayscale Digital Number and Radiation Intensity

**Figure 10:**
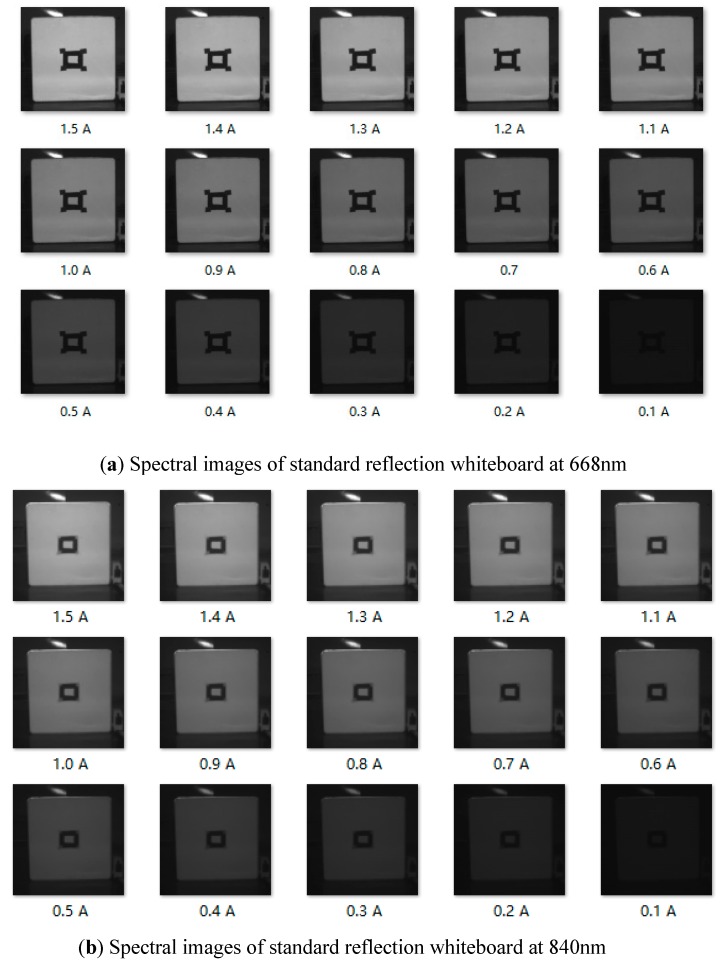
Spectral images of standard reflection whiteboard at 668nm and 840nm under different current drive.

After the incident radiation intensity is obtained, the intensity of the reflection is the key to calculating the reflectance. The Rededge-M multi-spectral camera can store 16-bit TIF digital format with a digital number of 65536. In order to calculate the radiation intensity of reflection, it is necessary to determine the relationship between the digital number (DN) and the radiation intensity of each point taken by the camera. For this purpose, the current of constant current power supply is adjusted to 0.1, 0.2, 0.3 to 1.5 A in darkroom to drive the active light source, and a standard reflective whiteboard with about 100% reflectivity is placed at 250–2500 nm with a distance of 1 m. The exposure time of the multi-spectral camera is set to 7.8 ms manually. In this test, Rededge-M multi-spectral camera is used to collect grey images of standard reflective whiteboard, and EU2000 + spectrometer is used to measure the radiation intensity of each adjustment of driving current. Spectral images of standard reflection whiteboard at 668nm and 840nm under different driving currents are shown in Figure 10.

**Figure 11:**
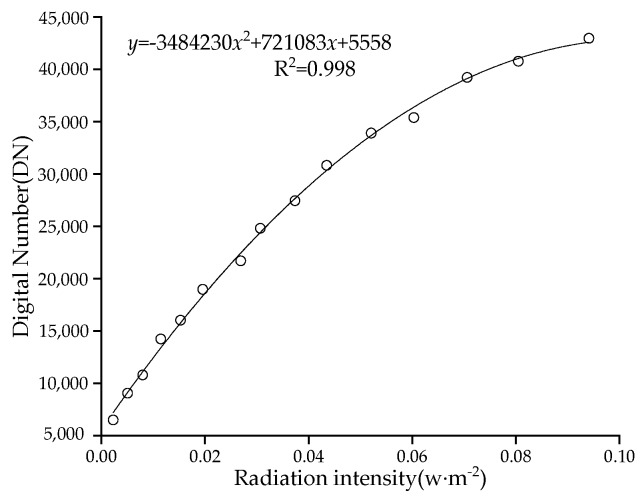
The relationship between the DN of the image display and the 668 nm red light radiation intensity.

**Figure 12:**
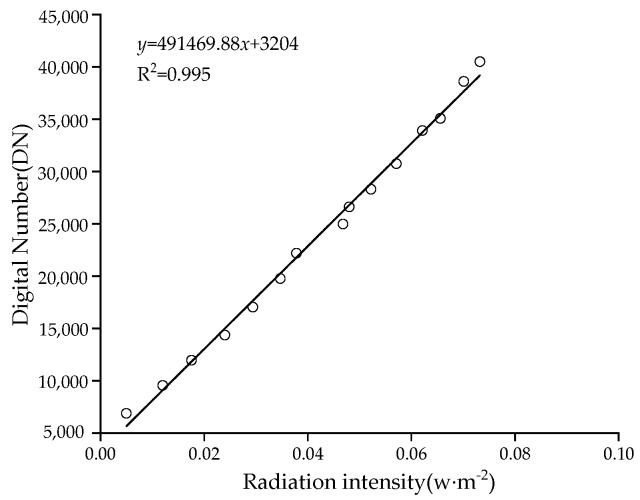
The relationship between the DN of the image display and the 840 nm infrared radiation intensity.

After the experiment, it is concluded that the display grey levels at 668 and 840 nm vary significantly with the change of driving current. The grey level DN of the multi-spectral camera has a quadratic regression relationship with the reflective radiation intensity at 668 nm (Figure 11), and a linear relationship with the reflective radiation intensity at 840 nm and the fixed exposure time (Figure 12). It can be seen from this that the radiation intensity of reflection can be calculated according to the grey level DN displayed in the image.

From Figure 11 and Figure 12, it can be seen that the 668 nm red light fitting curve is:

*y* = −3484230*x*^2^ + 721083*x* + 5558 (11)

The coefficient of determination R^2^ = 0.998. The 840 nm near-infrared light fitting curve is:

*y* = 491469.88*x* + 3204 (12)

### 3.5. Test Verification and Analysis

The GreenSeeker handheld crop sensor are often used to estimate the photosynthetic area or plant canopy nitrogen. Mature research and development techniques makes this device more accuracy in measuring the vegetation index. In order to verify the accuracy of NDVI index detection by active light source system, a validation experiment was carried out. Firstly, the multi-spectral camera is used to shoot the active light source at the distance of 2.85 m from the active light source, and the coordinates of four LED arrays in the spectral image are obtained. Then, 1.5 A constant current was used to drive the active light source, and the active light source was used to irradiate four potted plants placed 2.85 m away from the active light source. At the same time, the radiated potted plants were photographed with a multi-spectral camera.

**Figure 13:**
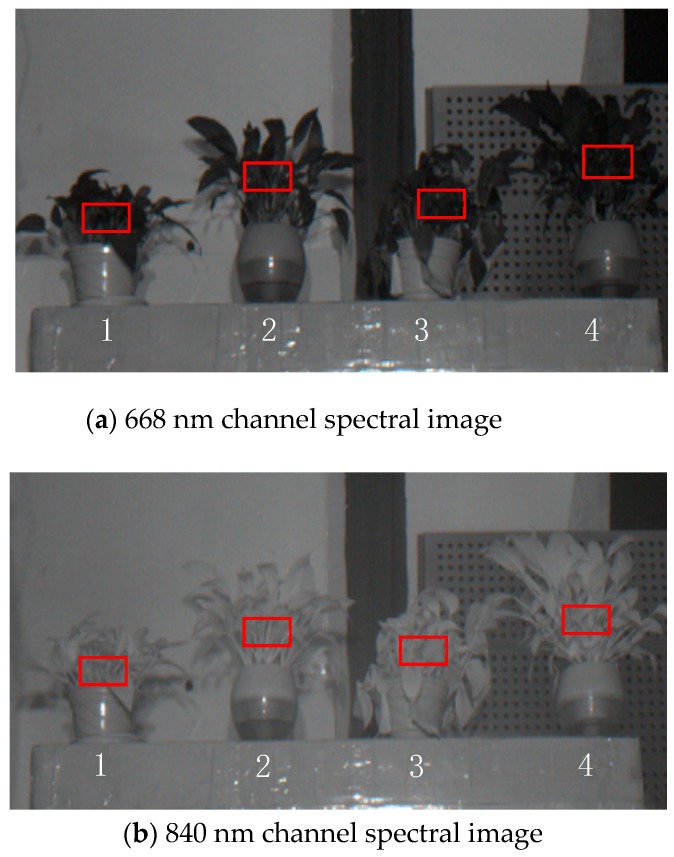
Multi-spectral photos with 2.85 m radiation range.

The center coordinates of active light source were 668_[1]_(−33,33), 668_[2]_(33,33), 840_[1]_(33,33), and 840_[2]_(33,33),and the spectral images are as shown in Figure 13. Numbers 1, 2, 3, and 4 with a red rectangle area are the representative areas. The average NDVI values of 10 samples measured by Greenseeker at 1 m were 0.534, 0.569, 0.680 and 0.610, respectively. When the distance was 2.85 m, the *Dx* =0.00025 m and pixel coordinates of 668_[1]_, 668_[2]_, 840_[1]_, and 840_[2]_ in the image were (32,32), (−32,32), (−32,−32) and (32,−32). The 668 nm average grayscale DN of the 4 red rectangles were 5178, 5291, 5255, and 5090. The grey-scale DN of 840 nm plant red rectangle in the image is 7411, 7576, 7891 and 8200, respectively. The coordinates of the four rectangles are 1 (−293,82), 2 (−108,111), 3 (64,92) and 4 (238,137).

According to Equations (9) and (10), the intensity of incident light can be obtained. According to the Equations (11) and (12), the intensity of reflected light radiation can be obtained. Then, according to the data in Table 8, NDVI was calculated by Equation (13). The correlation coefficient between NDVI value of active light source system and Greenseeker hand-held crop sensor is R = 0.995:

(13) $$NDVI_{\left(668,840\right)}=\frac{\frac{L_{ec}840}{L_{R}840}-\frac{L_{ec}668}{L_{R}668}}{\frac{L_{ec}840}{L_{R}840}+\frac{L_{ec}668}{L_{R}668}}$$

## 4. Discussion

In this paper, the difference between active and passive NDVI sensors in remote sensing is discussed, and their limitations are listed. The existing passive NDVI remote sensing technology mainly uses sunlight as the measuring light source, which depends heavily on the sunlight intensity and the zenith angle. The existing active NDVI remote sensing technology sensor has a small sensing area in one measurement and data from single measurements are scarce. Multi-spectral cameras can acquire a large amount of data in a single image acquisition, especially in remote sensing when carried by UAVs. In order to improve the efficiency of multi-spectral camera imaging technology, the combination of active non-parallel light source technology and multi-spectral imaging technology is proposed according to the respective advantages of active light source and spectral imaging technology. The basic characteristics of the active non-parallel light source and the optical attenuation caused by the geometrical structure of the imaging system are also considered. The attenuation of light will affect the quality of multi-spectral images and the correctness of final data. Because the light of the active light source in this paper is not parallel light, but a spherical light like scattered light, the radiation intensity of the vegetation surface is not equal. Therefore, it is necessary to measure and estimate the actual radiation intensity of vegetation sites, establish radiation attenuation model and radiation correction model to meet the radiation correction of non-parallel light.

We have conducted some experiments to verify The Inverse Square Law of light [30]. In other words, the radiation intensity of light decreases linearly with the square of distance. This theory is very important in the active remote sensing. Based on this optical rule and the geometric structure of the imaging system, the attenuation model and correction model of light are designed. In the attenuation model, we think that the radiation intensity of our light source is not enough. As can be seen from Figure 6, the radiation intensity decreases with the increase of distance. The longer the distance, the weaker the radiation intensity, even if the current is 2.5 A. As can be seen from Figure 7, at a distance of 2 m, the measured light radiation intensity is very weak, only about 0.02 W/m^2^. If long-distance light compensation is to be carried out, a higher power supply or a larger exposure time of the camera is required.

Our goal is to combine the active light source with the multi-spectral camera, and to take advantage of the advantages of the multi-spectral camera in collecting large amounts of data in an attempt to improve the operation time window. The light source we use here is a continuous light source, not a pulse light source, so the use of multi-spectral camera for photography and acquisition will still be affected by the external environment light. Because our experiments are carried out at night, the results and methods obtained are only suitable for testing without external light, but not for any light environment. If we want to use the combination of persistent light and multi-spectral cameras to obtain a larger remote sensing time window, we need to re-establish the radiation correction model, radiation attenuation model, and even the types of light sources in different lighting environments. In addition, a CW light source needs more power than a laser, so it is difficult for CW light source assistant technology to produce enough radiation intensity in multi-spectral camera remote sensing. Greenseeker’s NDVI is based on active laser pulse measurements and reflected sunlight measurements, so it should provide consistent readings no matter how the atmospheric or solar illumination conditions change [31]. A laser technology that can form a plane spot, such as vertical cavity surface emitting laser (VCSEL), is expected to be used in remote sensing of multi-spectral cameras. However, due to the limited response speed of the photosensitive elements of the multi-spectral camera to light and the image format compression technology required by the camera, it is difficult for the multi-spectral camera to measure the flash frequency of the laser source. This makes it difficult to filter out ambient light interference, even if the laser source can provide enough light intensity over a long distance. In the future, we believe that if we want to use active light source to achieve long-distance remote sensing monitoring, we need to further propose photosensitive technology.

## 5. Conclusions

In this paper, aiming at solving the problem of small working time window of passive NDVI sensor and small coverage area of single operation, we propose to combine multi-spectral imaging technology with active light source technology to collect the spectrum over a wide range. The results show that the intensity of radiation on the surface of different canopy points is different when radiated by a uniform light source. The main reason is that the distances between different points on the canopy surface and non-parallel light sources are different. In addition, the calculation factor of NDVI index is the reflectivity of infrared and near infrared light, so it is necessary to calibrate the incident light radiation at different points on the canopy surface. It is also necessary to analyse the correlation between the grayscale DN and the reflected radiation intensity value in the spectral image. We get the relationship between the grey-scale DN of red light and near-infrared light and the radiation intensity of reflection, and calculate the radiation intensity reflected from the measuring point according to the grey-scale DN. Finally, NDVI is obtained according to the intensity of reflected radiation and incident light. After four comparative experiments, the correlation coefficient between the proposed method and the results of the Greenseeker hand-held crop sensor professional spectral tester is 0.995, and the effect is remarkable. Although the system validates the feasibility of active spectral imaging technology in vegetation index detection without solar interference, if we want to apply this technology to UAV remote sensing, the radiation intensity and working distance of the light source need to be carefully considered. In the case of insufficient sunlight, the high sensitivity of the sensor and the intelligent supplementary light technology for obtaining high quality spectral images need to be further studied.

## Figures and Tables

**Table 1:** Main specifications of Rededge-M.

Parameter	Unit	Value
Weight	g	150 g
Dimensions	cm^3^	12.1 cm × 6.6 cm × 4.6 cm
Power	w	5.0 V DC, 4 w
Spectral bands		blue, green, red, red edge, NIR
Capture rate	s	1 s (max)
Format		16 bits TIFF, 12 bits DNG
Field of view	°	47.2°

**Table 2:** Spectral bands of the Rededge-M.

Band Number	Band	Center Wavelength (nm)	Band Width FWHM (nm)
1	Blue	475	20
2	Green	560	20
3	Red	668	10
4	Near IR	840	40
5	Red Edge	717	10

**Table 3:** Specifications of the constant current source.

Parameter	Unit	Value
Input voltage	V	100 ~ 240
Output voltage	V	26~36
Output current	mA	300 ~ 3200

**Table 4:** Main specifications of EU2000+ spectrometer.

Parameter	Unit	Value
Range of band	nm	400~1000
accuracy	nm	±0.4
Linearity of photometry	%	±0.5
Integration time	ms	3~60000
Sensor	/	2048 units CCD array
The optical fiber connector	/	SMA905

**Table 5:** Ratio of red light and infrared radiation intensity of different driving currents at twice the projection distance.

Twice Test Distance/cm	Current/ A	RIR _(668)_	RIR _(840)_
30/60	0.5	3.70	3.91
	1	3.55	3.91
	1.5	3.85	3.95
	2	3.65	4.22
	2.5	3.54	4.59
40/80	0.5	4.33	3.80
	1	3.78	3.38
	1.5	3.98	3.62
	2	3.98	4.13
	2.5	3.81	3.87
50/100	0.5	3.57	3.50
	1	4.07	4.06
	1.5	4.11	4.36
	2	4.13	3.73
	2.5	4.22	3.76

RIR _(668)_ means radiation intensity ratio at 668 nm (w/m^2^); RIR _(840)_ means radiation intensity ratio at 840 nm (w/m^2^).

**Table 6:** Coordinate value and radiation intensity.

Coordinate Label	Pixel Coordinate	Radiation Intensity(w/m^2^)
668_[1]_	($\frac{91}{L2}$, $\frac{91}{L2}$)	$\frac{L_{e}668}{L_{2}{}^{2}}$
668_[2]_	($-\frac{91}{L2}$, $\frac{91}{L2}$)	$\frac{L_{e}668}{L_{2}{}^{2}}$
840_[1]_	($-\frac{91}{L2}$, $-\frac{91}{L2}$)	$\frac{L_{e}840}{L_{2}{}^{2}}$
840_[2]_	($\frac{91}{L2}$, $-\frac{91}{L2}$)	$\frac{L_{e}840}{L_{2}{}^{2}}$

Le668 and Le840 are radiation intensity of one LED array when distance of EU2000+ spectrometer and LED array is 1 m; *L*_2_ > 1m.

**Table 7:** Predicted light intensity and measured light intensity.

Coordinate (m)	Pixel Coordinates	P _(668)_/P _(840)_, (w/m^2^)	M _(668)_/M _(840)_, (w/m^2^)	Error 668 nm/840 nm, (w/m^2^)
T_0_(0,0)	0, 0	0.0234/0.0208	0.0233/0.0207	0.0001/0.0001
T_1_(0.2,0.2)	138, 138	0.0230/0.0203	0.0228/0.0203	0.0001/0.0000
T_2_(-0.3,0.3)	−223, 223	0.0226/0.0197	0.0226/0.0199	0.0002/0.0003
T_3_(-0.4,0.4)	−283, 283	0.0219/0.0190	0.0216/0.0188	0.0003/0.0002
T_4_(0.1,0.1)	81, 81	0.0233/0.0206	0.0230/0.0204	0.0003/0.0002

P _(668)_ and P _(840)_ mean predicted radiation intensity (w/m^2^) based on attenuation correction model, respectively; M _(668)_ and M _(840)_ mean measured radiation intensity (w/m^2^) by the EU2000+ spectrometer, respectively.

**Table 8:** Test data comparison.

Number	IRI _(668nm)_	RRI _(668nm)_	IRI _(840nm)_	RRI _(840nm)_	NDVI(AS)	NDVI(GS)
1	0.01154	0.00365	0.01026	0.00856	0.449	0.534
2	0.01154	0.00370	0.01027	0.00926	0.475	0.569
3	0.01154	0.00368	0.01027	0.00953	0.488	0.580
4	0.01152	0.00361	0.01026	0.01015	0.519	0.610

IRI_(668)_ means incident radiation intensity at 668 nm (w/m^2^); IRI_(840)_ means incident radiation intensity at 840 nm (w/m^2^);RRI_(668)_ means reflected radiation intensity at 668 nm(w/m^2^); RRI_(840)_ means reflected radiation intensity at 840 nm(w/m^2^); NDVI(AS) means NDVI with Active light source; NDVI(GS) means NDVI with the GreenSeeker handheld crop sensor.
